# Association of sleep duration with chronic constipation among adult men and women: Findings from the National Health and Nutrition Examination Survey (2005–2010)

**DOI:** 10.3389/fneur.2022.903273

**Published:** 2022-08-10

**Authors:** Shuai Yang, Shou-Zhen Li, Fu-Zheng Guo, Dong-Xu Zhou, Xiao-Feng Sun, Jian-Dong Tai

**Affiliations:** ^1^Department of Ultrasound, The First Hospital of Jilin University, Changchun, China; ^2^Department of Colorectal and Anal Surgery, The First Hospital of Jilin University, Changchun, China

**Keywords:** sleep duration, constipation, cross-sectional study, stool consistency, NHANES

## Abstract

**Background:**

Previous studies suggested that unhealthy sleep patterns were closely associated with gastrointestinal diseases, but the impact of unhealthy sleep duration on chronic constipation has not been well studied until now. In this study, we aim to explore the association between sleep duration and constipation among males and females.

**Methods:**

We utilized the US National Health and Nutrition Examination Surveys data from 2005 to 2010, and adults (≥20 years old) who completed the sleep and bowel health questionnaires were enrolled in this observational study. Sleep duration was categorized into four groups: very short sleep (<5 h/night), short sleep (5–6 h/night), normal sleep (7–8 h/night), and long sleep (≥9 h/night). Chronic constipation was defined as Bristol Stool Scale Type 1(separate hard lumps, like nuts) or Type 2(sausage-like but lumpy). Controlling demographic, lifestyle, and dietary factors, the logistic regression model in Generalized Linear Model (GLM) function was used to estimate the correlation of sleep duration with constipation among men and women.

**Results:**

Of the 11,785 individuals (51.2% males and 48.8% females), 4.3% of men and 10.2% of women had constipation, respectively. More than half of patients with constipation did not adopt the recommended sleep duration. Compared with normal individuals, male participants with constipation had a higher proportion of shorter sleep duration (41.0 vs. 32.3% in the short sleep group and 6.3 vs. 4.7% in the very short sleep group), and female individuals with constipation had a higher proportion of long sleep duration (12.7 vs. 8.2%). After covariates adjustment, men with short sleep duration (5–6 h/night) correlated with increased odds for constipation (OR:1.54, 95%CI:1.05–2.25), and women with long sleep duration (≥9 h/night) linked to the higher constipation risk (OR:1.58, 95%CI:1.10–2.29). Excessive sleep duration in males or insufficient sleep duration in females was neither linked to increased nor decreased constipation risk.

**Conclusions:**

In this observational study of a nationally representative sample of adults, we demonstrate a differential impact of unhealthy sleep duration on constipation among men and women. Short sleep duration poses a higher risk of constipation in men, and excessive sleep duration correlates with higher constipation risk in women.

## Introduction

Sleep disorders affect many individuals worldwide and is increasing alarmingly (1). Both short and excessive sleep duration may contribute to a lower quality of life and unfavorable health outcomes. Plenty of evidence has already confirmed their association with a higher risk of cardiovascular diseases, stroke, type 2 diabetes, and mortality (2). However, the impact of sleep quality on gastrointestinal disorders is often underappreciated in clinical practice. Over the past years, emerging studies have indicated that unhealthy sleep patterns can contribute significantly to the pathogenesis of gastroesophageal and small intestinal disorders (1, 3–5). People with frequent sleep disturbance are more likely to experience peptic ulcer disease, gastroesophageal reflux disease, and irritable bowel syndrome because of the disruption of circadian rhythms and immune systems (4). Its influence on colorectal function has rarely been elucidated.

Chronic constipation is a highly prevalent gastrointestinal disorder that affects about 10.1–15.3% of the adult population globally, characterized by hard stools, infrequent bowel movements, excessive straining, abdominal pain, and the feeling of incomplete evacuation (6, 7). Various methods are currently adopted to define constipation. Besides the gold standard of the Rome criteria, considering clinical applicability, Markland suggested that the stool consistency described by Bristol Stool Form Scale (BSFS) was also a validated way to diagnose chronic constipation because of its high correlation with colon transit time (8). Many risk factors linked to chronic constipation have already been reported, including dietary intake, age, drug use, the dysfunction of colonic propulsion or rectal emptying, dysbacteriosis, and metabolic disorder (9–12). However, minimal research has investigated the correlation between sleep duration during the night and chronic constipation among adult males and females (13). We hypothesize that inappropriate sleep duration at night may pose a higher risk of constipation in both men and women.

This observational research aims to evaluate the correlation of sleep duration with chronic constipation among women and men with adjustment for other possible confounding factors, including lifestyle and demographic characteristics and dietary intakes such as energy, fiber, and moisture, by using the National Health and Nutrition Examination Survey (NHANES) database.

## Materials and methods

### Study population

**Figure 1:**
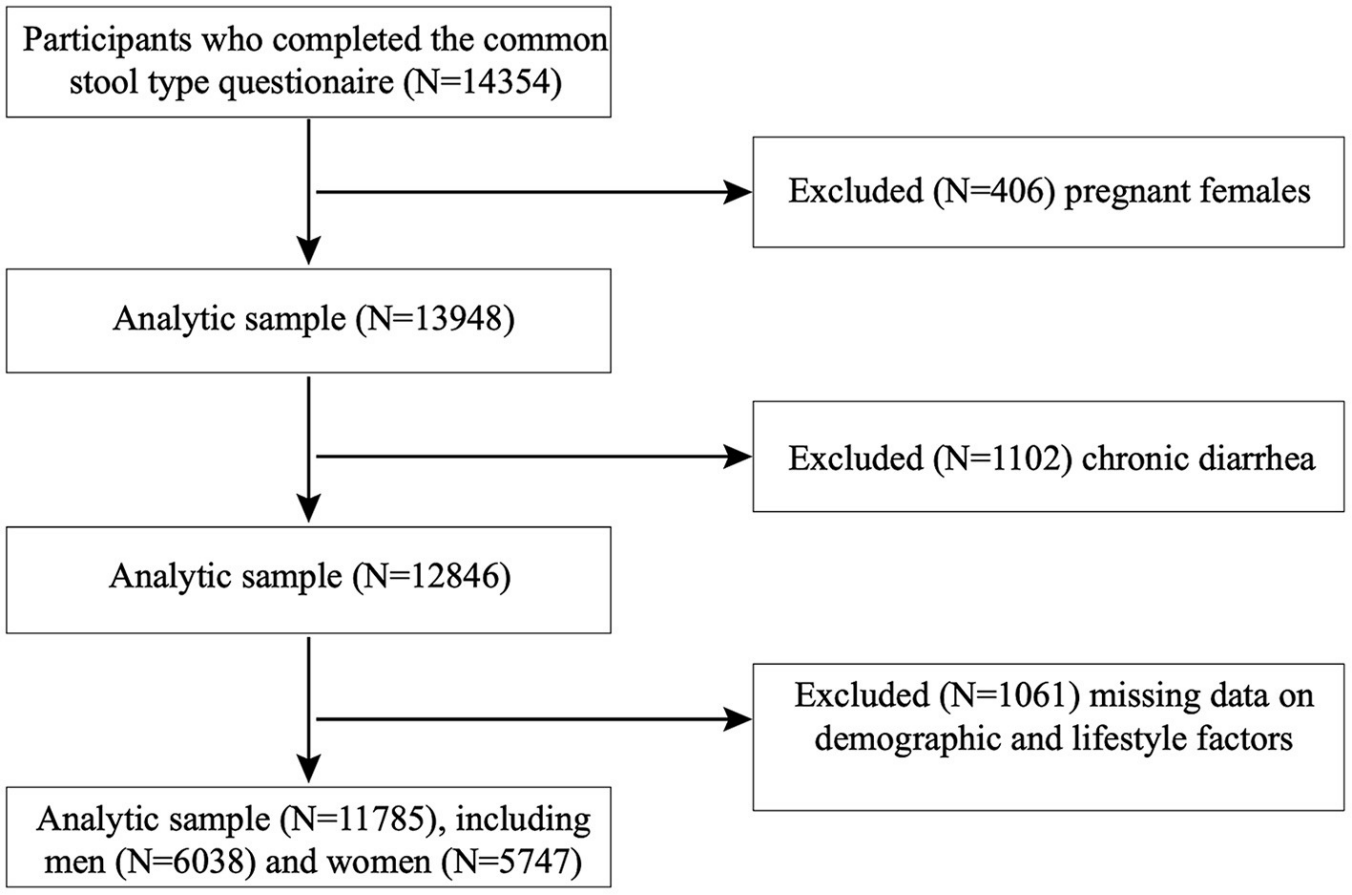
Flow chart of the selection process.

Participants in this cross-sectional analysis were included from the NHANES 2005–2010, which was carried out by the Centers for Disease Control and Prevention (CDC) in the United States. Using a stratified, multistage, probability cluster design, all health-related data obtained in this program represented the general non-institutionalized United States population. All individuals provided written informed consent, which was approved by the ethics review board of the CDC. We included 14354 adults aged 20 years or older who completed the common stool type questionnaire and sleep disorder questionnaire. We excluded pregnant women (*N* = 406) and participants with chronic diarrhea (*N* = 1,102) and missing information on demographic data (*N* = 924) and other possible covariates (diabetes, smoking, drinking, hypertension, dietary intake, *N* = 137). Therefore, a final sample of 11,785 adults (6,038 men and 5,747 women) was included in our analysis (Figure 1).

### Sleep duration

The trained interviewer administered sleep disorders in the home using the Computer-Assisted Personal Interviewing-CAPI system. Sleep duration information was obtained based on the question, “how much sleep is usually getting at night on weekdays or workdays?” Sleep duration was classified into four groups: very short sleep (<5 h/night), short sleep (5–6 h/night), normal sleep (7–8 h/night), and long sleep (≥9 h/night). 7–8 h per night was defined as the reference group (14–16).

### Definition of constipation

Given the former research that estimated constipation in the NHANES surveys, using the stool consistency described by Bristol Stool Form Scale (BSFS) to define constipation has been regarded as a validated measure. Stool consistency information was recorded in the bowel health questionnaire for three cycles of NHANES 2005–2010. Participants were shown a colored card with descriptions of stool types and confirmed the usual or most common stool type. BSFS Type 1 (separate hard lumps like nuts) or Type 2 (sausage-like but lumpy) was regarded as chronic constipation. BSFS Type 6 (fluffy pieces with ragged edges, a mushy stool) or Type 7 (watery, no solid pieces) was considered chronic diarrhea. BSFS Type 3, Type4, and Type 5 (like a sausage but with cracks in the surface, smooth and soft, or soft blobs with clear-cut edges) were regarded as normal stool consistency.

### Covariates

The demographic characteristics in the analysis included age, race and ethnicity (Mexican American, non-Hispanic white, non-Hispanic black, and other races), educational attainment (< high school, high school, > high school levels), marital status (married or living with a partner and living alone), and family poverty income ratio (family income was categorized into two groups: <2.0, ≥2.0). Body mass index (BMI) was grouped into the following classifications: underweight or normal (<25 kg/m^2^), overweight (25–30 kg/m^2^), and obese (>30 kg/m^2^). Lifestyle factors included alcohol use (Drinking status was regarded as positive if participants responded to “yes” to the question of “Had at least 12 alcohol drinks/1 year?”.), smoking status (if participants had smoked at least 100 cigarettes in their entire life was considered as positive), diabetes (no diabetes, having diabetes, borderline), hypertension (if participant been told by a health professional that had high blood pressure was regarded as positive). The dietary intake such as energy, protein, fiber, saturated fatty acid, polyunsaturated fatty acid and moisture (water intake including liquid from foods and beverages) among men and women were obtained from the two 24-h dietary recall interviews and averaged.

### Statistical methods

All data analysis considered the weighted and clustered sampling design of NHANES, and the new 6-year weight was reweighted (1/3 of the 2005–2010 weight) according to the NHANES guidelines. Continuous variables were described by mean and 95% confidence intervals (CIs), and categorical variables were described by survey-weighted percentage (95% CI). The survey-weighted Chi-square test tested the difference between categorical variables. The weighted logistic regression model in Generalized Linear Model (GLM) function was performed to compute the odds ratios (ORs) and 95% CIs of the correlation between sleep duration and chronic constipation in men and women, with normal sleep as reference. Three different logistic models were built; model 1 adjusted for age, race and ethnicity, and educational attainment, marital status, and family poverty income ratio. Model 2 controlled age, race and ethnicity, educational attainment, marital status, drinking status, smoking status, diabetes status, hypertension, BMI, and family poverty income ratio. Model 3 controlled for variables in model 2 and dietary intakes such as energy, protein, fiber, cholesterol, saturated fatty acids, polyunsaturated fatty acids, monounsaturated fatty acids and moisture. All analyses were performed with the statistical software R (the R Foundation; http://www.r-project.org; version 3.4.3 2021-12-21) and Empower R (www.empowerstats.com, X&Y, solutions, inc.), *P*-value <0.05 (two-sided) was regarded as a statistical significance.

## Results

### Characteristics of participants

The baseline characteristics of participants with or without constipation were presented in Table 1. Of the 11785 individuals (51.2% males and 48.8% females), 4.3% of men and 10.2% of women had constipation, respectively. Compared with normal individuals, male participants with constipation were associated with shorter sleep duration (41.0 vs. 32.3% in the short sleep group and 6.3 vs. 4.7% in the very short sleep group), and female individuals with constipation had a higher proportion of long sleep duration (12.7 vs. 8.2%). Statistical differences in race and ethnicity prevalence were also observed in the study. Male participants with constipation were also more likely to have lower educational levels, living alone, have less family poverty income ratio, have lower BMI, shorter sleep duration, and less dietary intakes such as protein, fiber, and moisture. Among women, other factors correlated with constipation on univariate analysis were lower educational levels, lower family incomes, lower BMI, no drinking, less dietary intake of fiber, polyunsaturated fatty acids, and moisture.

**Table 1:** Participant characteristics by constipation among men and women from NHANES 2005–2010.

**Characteristics**	**Male**, ***N** =* **6038**		***P*** **-value**	**Female**, ***N** =* **5747**		***P*** **-value**
	**No constipation** **(*N =* 5726)**	**Constipation** **(*N =* 312)**		**No constipation** **(*N =* 5109)**	**Constipation** **(*N =* 638)**	
Age, years	45.8 (45.0, 46.6)	44.5 (41.9, 47.0)	0.296	47.7 (46.9, 48.5)	46.4 (44.9, 47.9)	0.131
Race/ethnicity, % (95% CI)^a^			<0.001			0.012
Mexican American	8.1 (6.4, 10.2)	16.6 (11.7, 23.0)		6.5 (5.0, 8.3)	7.2 (5.3,9.8)	
Non-Hispanic white	72.9 (69.2, 76.2)	57.9 (48.2, 67.0)		74.5 (70.7, 77.9)	68.1 (60.9,74.6)	
Non-Hispanic black	9.9 (8.3, 11.8)	17.3 (12.4, 23.7)		10.6 (8.7, 12.8)	14.5 (10.6, 19.5)	
Others	9.1 (7.6, 10.9)	8.3 (4.4, 15.1)		8.5 (7.1, 10.2)	10.2 (7.0, 14.6)	
Educational attainment % (95% CI)			<0.001			<0.001
< High school	16.7 (15.0, 18.6)	30.1 (23.5, 37.7)		16.0 (14.1, 18.1)	21.3 (17.5, 25.7)	
High school	24.4 (22.5, 26.5)	32.3 (25.4, 40.1)		23.6 (22.0, 25.3)	27.8 (23.1, 33.1)	
>High school	58.9 (55.9, 61.8)	37.6 (29.0, 47.1)		60.4 (58.50, 62.8)	50.4 (44.6,56.1)	
Marital status, % (95% CI)			0.032			0.193
Married or living with partner	67.3 (65.1, 69.5)	58.3 (49.7, 66.3)		61.0 (58.3, 63.7)	57.3 (51.8, 62.6)	
Living alone	32.7 (30.5, 34.9)	41.7 (33.7, 50.3)		39.0 (36.3, 41.7)	42.7 (37.4, 48.2)	
Family poverty income ratio			<0.001			<0.001
<2.0	29.6 (27.5,31.9)	43.9 (37.0,51.0)		33.1 (30.8, 35.4)	42.4 (37.0, 48.0)	
≥2.0	70.4 (68.1,72.5)	56.1 (49.0,63.0)		66.9 (64.6, 69.2)	57.6 (52.0, 63.0)	
BMI**(**kg/m^2^**)**, % (95% CI)			0.030			0.017
<25kg/m^2^	26.1 (23.9, 28.4)	34.8 (26.4, 44.3)		36.2 (34.2, 38.4)	41.3 (35.8, 47.0)	
25–30kg/m^2^	39.2 (37.5, 40.9)	37.8 (30.9, 45.2)		28.4 (26.2, 30.7)	31.5 (26.7, 36.9)	
>30kg/m^2^	34.7 (32.4, 37.2)	27.5 (21.8, 33.9)		35.4 (33.4, 37.4)	27.2 (23.1, 31.7)	
Drinking status, % (95% CI)			0.086			0.002
Yes	86.0 (84.3, 87.6)	81.8 (76.7, 86.0)		69.4 (66.6, 72.0)	61.3 (55.5, 66.8)	
No	14.0 (12.4, 15.7)	18.2 (14.0, 23.3)		30.6 (28.0, 33.4)	38.7 (33.2, 44.5)	
Smoking status, % (95% CI)			0.540			0.510
Yes	53.1 (50.8,55.4)	55.4 (47.5,63.1)		42.0 (39.8,44.3)	37.1 (32.2,42.2)	
No	46.9 (44.6,49.2)	44.6 (36.9,52.5)		58.0 (55.7,60.2)	62.9 (57.8,67.8)	
Diabetes status, % (95% CI)			0.596			0.105
Yes	7.5 (6.8, 8.3)	6.8 (4.1, 10.8)		7.2 (6.3, 8.2)	9.0 (6.8, 11.7)	
No	90.7 (89.8,91.5)	92.4 (88.0, 95.3)		91.3 (90.1, 92.4)	90.3 (87.7, 92.4)	
Borderline	1.8 (1.4, 2.3)	0.9 (0.2, 4.8)		1.5 (1.2, 1.9)	0.8 (0.3, 1.8)	
Hypertension, % (95% CI)			0.207			0.616
Yes	29.3 (27.4,31.4)	25.2 (19.2,32.3)		31.3 (29.4, 33.3)	30.1 (25.6, 35.1)	
No	70.7 (68.6,72.6)	74.8 (67.7,80.8)		68.7 (66.7, 70.6)	69.9 (64.9, 74.4)	
Sleep duration			0.047			0.006
Very short sleep	4.7 (4.0,5.5)	6.3 (3.4,11.5)		5.1 (4.3,6.1)	5.9 (4.3,8.2)	
Short sleep	32.3 (30.8,33.9)	41.0 (32.3,50.3)		28.8 (26.8,30.8)	30.5 (26.3,35.1)	
Normal sleep	57.0 (55.2,58.8)	46.8 (39.0,54.8)		57.9 (55.4,60.2)	50.9 (45.5,56.2)	
Long sleep	6.0 (5.2,6.8)	5.9 (3.4,10.0)		8.2 (7.2,9.4)	12.7 (9.7,16.3)	
Energy intake **(**kcal/d**)**	2,516.8 (2,476.9, 2,556.7)	2,350.1 (2,219.6, 2,480.6)	0.019	1,769.1 (1,745.4, 1,792.9)	1,714.2 (1,659.9, 1,768.5)	0.057
Protein intake (gm/d)	89.0 (87.6, 90.4)	81.6 (76.7, 86.4)	0.007	75.5 (74.5, 76.5)	74.7 (71.2, 78.2)	0.612
Fiber intake (gm/d)	17.2 (16.9, 17.5)	16.0 (14.9, 17.1)	0.024	15.9 (15.7, 16.2)	15.2 (14.6, 15.8)	0.018
Saturated fatty acids intake (gm/d)	28.1 (27.6, 28.7)	26.2 (24.4, 28.0)	0.052	23.9 (23.5, 24.3)	23.2 (22.0, 24.3)	0.245
Monounsaturated fatty acids intake (gm/d)	31.4 (30.8, 31.9)	30.0 (28.2, 31.8)	0.147	26.5 (26.1, 26.9)	25.3 (24.3, 26.4)	0.065
Polyunsaturated fatty acids intake (gm/d)	18.4 (18.0, 18.7)	17.1 (16.1, 18.1)	0.022	16.0 (15.7, 16.3)	15.0 (14.5,15.5)	0.007
Cholesterol intake (mg/d)	320.8 (314.0, 327.5)	283.7 (260.5, 306.9)	0.003	262.7 (256.9, 268.5)	262.6 (245.3, 280.0)	0.996
Moisture intake (gm/d)	3,007.5 (2,965.6, 3,049.3)	2,801.7 (2,666.5, 2,937.0)	0.004	2,755.7 (2,715.5, 2,795.9)	2,606.6 (2,501.9, 2,711.2)	0.005

a Survey-weighted percentage (95% CI); P-value: survey-weighted Chi-square test.

### Multivariable analyses of risk factors for constipation

**Figure 2:**
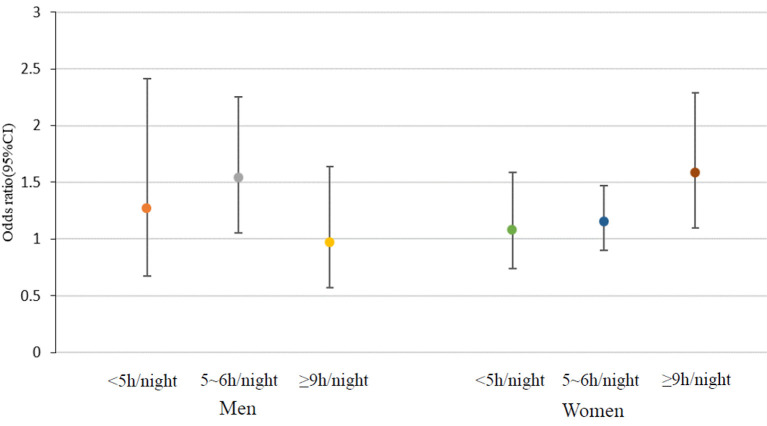
Association of sleep duration with constipation in men and women.

Table 2 presented the weighted ORs (95% CI) of constipation based on sleep duration among men and women. The short sleep duration (5–6 h/night) remained linked to enhanced constipation risk after covariates adjustment in different models among males. The ORs(95%CI) of constipation for that level of sleep duration, compared with the normal group, was 1.49(1.03–2.17) in model 1(adjusted for age, educational attainment, race and ethnicity), 1.53(1.04–2.24) in model 2(controlled for covariates in model 1, and also family poverty income ratio, drinking, smoking, diabetes, hypertension, BMI), and 1.54(1.05–2.25) in model 3 (controlled for confounders in model 2, and also dietary intakes such as energy, protein, and moisture). Long sleep duration (≥9 h/night) among females was associated with a higher risk of chronic constipation. The weighted ORs(95%CI) of constipation for that group was 1.67(1.16–2.41) in model 1, 1.59(1.11–2.30) in model 2, 1.58(1.10–2.29) in model 3. Other sleep duration groups in both men and women did not show a significant correlation with constipation (Figure 2).

**Table 2:** weighted ORs and 95%CIs for constipation among men and women according to the sleep duration.

	**Model 1** **OR(95%CI)**	**Model 2** **OR(95%CI)**	**Model 3** **OR(95%CI)**
**Men**			
normal	1.00 (ref.)	1.00 (ref.)	1.00 (ref.)
<5 h/night	1.23 (0.66–2.31)	1.29 (0.68–2.47)	1.27 (0.67–2.41)
5–6h/night	1.49 (1.03–2.17) ^*^	1.53 (1.04–2.24) ^*^	1.54 (1.05–2.25) ^*^
≥9h/night	1.04 (0.61–1.78)	1.03 (0.61–1.74)	0.97 (0.57–1.64)
**Women**			
Normal	1.00 (ref.)	1.00 (ref.)	1.00 (ref.)
<5h/night	1.14 (0.79–1.66)	1.11 (0.74–1.65)	1.08 (0.74–1.59)
5–6h/night	1.14 (0.89–1.46)	1.15 (0.90–1.46)	1.15 (0.90–1.47)
≥9h/night	1.67 (1.16–2.41) ^*^	1.59 (1.11–2.30) ^*^	1.58 (1.10–2.29) ^*^

OR, odds ratio; CI, confidence interval. Model 1 controlled for age, race/ethnicity, educational attainment, marital status, family poverty income ratio; Model 2 adjusted for age, race/ethnicity, educational attainment, marital status, family poverty income ratio, drinking, smoking, diabetes status, hypertension, BMI; Model 3 controlled for age, race, educational attainment, marital status, family poverty income ratio, drinking, smoking, diabetes status, hypertension, BMI and dietary intake of energy, protein, fiber, saturated fatty acids, monounsaturated fatty acids, polyunsaturated fatty acids, cholesterol, and moisture.

* P < 0.05.

## Discussion

This population-based observational analysis found that nearly half of the population did not adopt the recommended sleep duration. More than one-third of people reported sleeping <7 h per night, and females have a higher prevalence of long sleep duration than males. In multivariable analyses, sleep duration at night was observed to be independently associated with chronic constipation among men and women. An estimated sleep duration of 5–6 h/night correlated with enhanced risk of constipation in men, excessive sleep durations (≥9 h/night) were linked to elevated constipation risk in women, and insufficient sleep time in women or excessive sleep time in men would not change the prevalence of chronic constipation.

To our knowledge, this is the first research to investigate the independent correlation of sleep duration with chronic constipation with a nationally representative sample. Most previous studies have estimated the correlation between sleep disorders and gastrointestinal conditions. Plenty of evidence confirmed that sleep disorders correlated with a higher risk of functional gastrointestinal disorders like reflux and functional constipation, even with severity (17–22). However, the effect of dimensions of sleep status such as sleep latency and sleep duration has been finitely reported until now. In the sample of adolescents, the relationship between sleep time and functional constipation has been regarded as no significant, and for children, insufficient sleep duration could increase the risk of constipation (23, 24), but little research on the adult population. Our results confirmed a differential relationship between sleep duration and chronic constipation among men and women.

Several underlying pathways could explain the observed correlation between short sleep duration and chronic constipation. Firstly, short bedtime may change the circadian rhythms of gastrointestinal physiology, readjust sleep phase proportion, and enhance nocturnal wake frequency, causing abnormal increased basal colonic motility and contraction in sleep periods compared with colonic activity decrease in healthy individuals (22). In the daytime, in patients with insufficient sleep, the food intake may not induce the normal increase of distal colonic cyclic propagating motor activity like in healthy adults (25, 26). Secondly, short sleep duration would alter the rapid eye movement (REM) sleep phase, which is believed that disturbance could predispose to the development of abnormal bowel gastrointestinal transit, supported by abnormal α-synuclein immunoreactivity in colonic submucosal nerve fibers or ganglia and expression and function of alpha2A-adrenoceptor in the distal ileum (27, 28). Thirdly, emerging evidence from experimental research showed that short sleep duration might enhance vulnerability to constipation by altering average levels of inflammatory markers such as interleukin-6 and C-reactive protein (29, 30).

While emerging evidence reveals the negative health implications of insufficient sleep on gastrointestinal function, few studies pay attention to the risks of excessive sleep. Our results indicated that long sleep time could also enhance the risk of chronic constipation in women. Possible explanations are as follows: Firstly, prolonged sleep time may increase sleep fragmentation which is thought to be correlated with induced microbial dysbiosis and exacerbation of enteric symptoms, just as patients with irritable bowel syndrome (IBS) do (31, 32). Next, excessive sleep duration always means decreased physical activity, an essential factor associated with a reduced risk of constipation (33, 34). In addition, the indirect evidence from a meta-analysis indicates that excessive sleep time is significantly associated with increased depression risk, and depressed condition and functional constipation often co-occur and coexist in the clinic (35, 36). Fourthly, the differential result among men and women reveals that abnormal sleep duration may also affect an intermediate factor, estrogen level, to impact further bowel movement, confirmed in animal experiments (37).

Our research has several strengths. Firstly, we evaluate the correlation between sleep duration and constipation among males and females using a nationally representative sample for the first time. Secondly, the effect of short and long sleep duration on constipation has been assessed. Thirdly, we adjusted possible confounding factors in the analyses. Several limitations also should be considered. Due to the cross-sectional study nature, we cannot determine the causality between sleep duration and functional constipation. Secondly, we use the BSFS instead of the gold standard of the Rome criteria to define constipation may underestimate the prevalence of constipation. Thirdly, we only focus on the effect of different sleep duration on constipation, but the sleep condition and sleep quality have not been well considered. The causes of sleep disorders differ between men and women, for instance, sleep apnea is more common in males while insomnia is more common in males, so additional studies are needed to explore definite causes and mechanisms. Fourthly, sleep duration in the NHANES database is measured by retrospective self-report rather than gold-standard polysomnography. Therefore, the recall bias may occur.

In future work, it is necessary to investigate the causal relationship between sleep disorders and chronic constipation with randomized controlled trials. Next, we should focus more on the sleep quality of patients with constipation and the concrete causes of sleep disorders. In addition, the colonic changes such as motor patterns and pathology in constipation patients with sleep disorders should be investigated.

## Conclusions

Unhealthy sleep duration independently correlates with a higher risk of constipation based on the NHANES data from 2005 to 2010. Short sleep duration can increase chronic constipation risk in men, and excessive sleep duration is associated with the risk of constipation in women. We believe that discovery provides new insight into the long-term management of chronic constipation.

## Data availability statement

Publicly available datasets were analyzed in this study. This data can be found here: Centers for Disease Control and Prevention (CDC) National Health and Nutrition Examination Survey (NHANES), https://wwwn.cdc.gov/nchs/nhanes/Default.aspx, 2005–2010.

## Ethics statement

The studies involving human participants were reviewed and approved by the Ethics Review Board of the NCHS. The patients/participants provided their written informed consent to participate in this study.

## Author contributions

X-FS and SY designed the study. S-ZL and F-ZG acquired the data. S-ZL and D-XZ analyzed the data. SY drafted the manuscript. X-FS and J-DT critically revised the manuscript. All authors read and approved the final manuscript.

## Conflict of interest

The authors declare that the research was conducted in the absence of any commercial or financial relationships that could be construed as a potential conflict of interest.

## Publisher's note

All claims expressed in this article are solely those of the authors and do not necessarily represent those of their affiliated organizations, or those of the publisher, the editors and the reviewers. Any product that may be evaluated in this article, or claim that may be made by its manufacturer, is not guaranteed or endorsed by the publisher.
